# Defining the impaction frequency and threshold force required for femoral impaction grafting in revision hip arthroplasty: A human cadaveric mechanical study

**DOI:** 10.3109/17453674.2011.594228

**Published:** 2011-09-02

**Authors:** Fionnan Cummins, Peter O' Reilly, Olivia Flannery, Danny Kelly, Paddy Kenny

**Affiliations:** ^1^Trinity Centre for Bioengineering, School of Engineering, Trinity College Dublin; ^2^Department of Orthopaedics, Cappagh National Orthopaedic Hospital and Connolly Hospital, Dublin, Ireland; Correspondence: fionnanc@gmail.com

## Abstract

**Background and purpose:**

The two most common complications of femoral impaction bone grafting are femoral fracture and massive implant subsidence. We investigated fracture forces and implant subsidence rates in embalmed human femurs undergoing impaction grafting. The study consisted of two arms, the first examining the force at which femoral fracture occurs in the embalmed human femur, and the second examining whether significant graft implant/subsidence occurs following impaction at a set force at two different impaction frequencies.

**Methods:**

Using a standardized impaction grafting technique with modifications, an initial group of 17 femurs underwent complete destructive impaction testing, allowing sequentially increased, controlled impaction forces to be applied until femoral fracture occurred. A second group of 8 femurs underwent impaction bone grafting at constant force, at an impaction frequency of 1 Hz or 10 Hz. An Exeter stem was cemented into the neomedullary canals. These constructs underwent subsidence testing simulating the first 2 months of postoperative weight bearing.

**Results:**

No femurs fractured below an impaction force of 0.5 kN. 15/17 of the femurs fractured at or above 1.6 kN of applied force. In the second group of 8 femurs, all of which underwent femoral impaction grafting at 1.6 kN, there was no correlation between implant subsidence and frequency of impaction. Average subsidence was 3.2 (1–9) mm.

**Interpretation:**

It is possible to calculate a force below which no fracture occurs in the embalmed human femur undergoing impaction grafting. Higher impaction frequency at constant force did not reduce rates of implant subsidence in this experiment.

With the increasing success of acetabular impaction bone grafting in dealing with loss of acetabular bone stock, attempts were made to use the technique to fill femoral bone defects in revision hip arthroplasty. The results of femoral impaction grafting have been satisfactory. In a recent review of over 1,000 femoral impaction graftings, Ornstein et al. (2009) reported 15-year implant survival rates of 94%, with minimal difference in implant survivorship between low- and high-volume units, suggesting that the technique of femoral impaction grafting appears to be, “reliable, can be learned rapidly, and produces a predictably low incidence of aseptic loosening.”

Despite these results, however, there is still concern regarding the high rate of complications of femoral impaction grafting, the main complications being operative or perioperative femoral fracture and implant subsidence. Fracture rates of up to 16% have been reported (Masterson et al. 1997, Leopold et al. 1999, Pekkarinen et al. 2000). High rates of implant subsidence have also been described (Eldridge et al. 1997, Masterson et al. 1997), which may be due to inadequate impaction of the morselized bone graft. Thus, increasing the impaction force will ensure improved graft stability but will increase the risk of femoral fracture.

In a preliminary study on sow femurs, Flannery et al. (2010) achieved a stable construct without fracture. A stable construct was defined as a femur that underwent impaction bone grafting at sub-threshold force, with a cemented Exeter stem that did not undergo massive early subsidence (10 mm) on initial subsidence testing simulating the first 2 postoperative months of weight bearing. The authors were unable to find any correlation between threshold force, bone mineral density, cortex-to-canal ratio, or cortical thickness in impaction bone grafting in the adult sow femur.

In this study, we applied the experimental protocol of Flannery et al. (2010) to a sample of adult human femurs and investigated the above associations—but with the addition of measurement of cortex-to-canal ratios on standardized pretesting plain radiographs.

The compaction of the bone graft may also be dependent on the frequency of impaction. In a laboratory study on impacted pig bone, Marck Van Liefland (2006) reported that “high-frequency impaction achieved high compaction at low load.” in a pot of morselized pig bone graft. While high compaction was achieved, the author did not state how far the graft had compressed. “Compared to traditional impaction, the same amount of compaction was achieved at 10–20% of the load. Inversely, compaction almost doubled at the same load.” Thus, prevention of massive early subsidence of the femoral component may also be dependent on the frequency of impaction to obtain adequate compaction, possibly permitting lower forces to be applied at higher frequencies and possibly reducing fracture risk.

A final subset of femurs then underwent impaction bone grafting at set impaction force. This was followed by subsidence testing with a cemented Exeter stem, half at an impaction frequency of 1 Hz and the other half at an impaction frequency of 10 Hz.

## Materials and methods

Adult human embalmed femurs with no signs of surgery or fracture were obtained from the anatomy department of the Royal College of Surgeons in Ireland. The femurs was disarticulated at the knee and stripped of soft tissue. A second incision over the greater trochanter was sometimes necessary to dissect the hip joint capsule and labrum before disarticulation. The femoral heads were amputated as for hip replacement, and the femoral shafts were cut 17.5 cm from the neck cuts. The femurs then underwent a single AP radiograph of the femur. Cortex:canal measurements were made at 3 levels (top of lesser trochanter, middle of lesser trochanter, and base of lesser trochanter), and average measurements were used to calculate cortex:canal ratios. The femurs then underwent DEXA scanning, with calculation of an overall BMD and a BMD for each Gruen zone excluding Gruen zone 4.

22 human femurs were used. Of these, 17 underwent complete destructive testing. Of the 5 femurs that were not tested fully, the first 2 were broken prematurely by use of an inappropriately long proximal impactor, 2 femurs had an abnormally large sub-trochanteric saggital curve that precluded full insertion of the smaller proximal impactor, and 1 was accidentally fractured while clearing out the cortical canal with a hand reamer. The first 2 femurs to be fractured, fractured with maximum strain recorded at the distal Gruen zones, suggesting that the distal tip of the impactor was levering on the bone. These 2 were excluded from the analysis. A second impactor was used for the remaining experiments with maximum strain recorded at the proximal Gruen zones.

The femoral canal was then cleared, leaving only a cortical shell, and the femur was potted upright in a plug of dental simplex cement. Strain gauges were then applied according to Dabestini (1991) with some modifications. The strain gauges were applied medially, laterally, and anteriorly on the femurs at two levels; at the level the junction between Gruen Zones 1 and 2, and 6 and 7 and lower at the junction of Gruen Zones 2 and 3, and 5 and 6.

**Figure 1.**
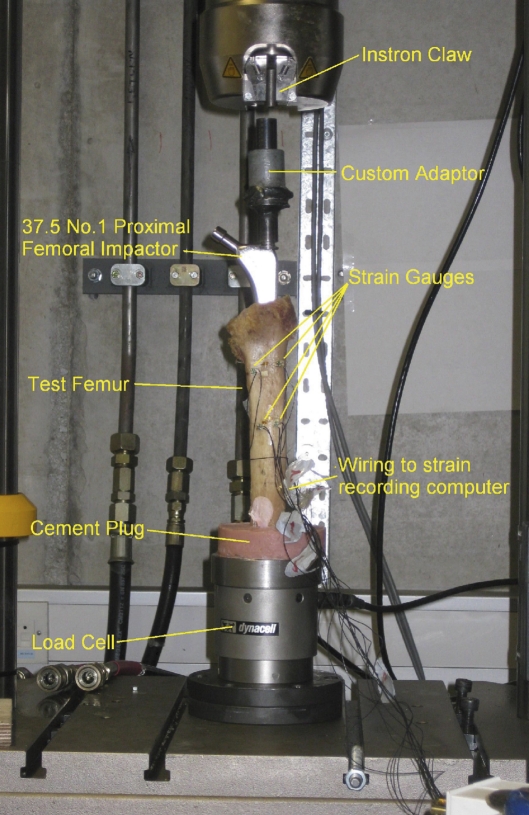
Impaction using the X-Change III proximal femoral impactor size 37.5 (1) attached to a biaxial hydraulic fatigue-testing machine.

The excised femoral heads, medullary material, and femoral condyles were morselized and used as graft. Ronguers/bone nibblers with an optimal bite of 10 mm were used to morselize the bone graft as recommended by Schreurs et al. (1998). A variety of chip sizes was obtained using the rongueurs as in the intraoperative situation. A variety of chip sizes results in a mechanically stronger graft construct (Brewster al. 1999). Bone graft was introduced into the distal canal and compressed by hand using a steel rod. This was repeated until the bone graft had reached a level 10 cm from the tip of the greater trochanter. The X-Change III size 1, 37.5-mm offset proximal impactor (Stryker-Howmedica, Berkshire, UK) was then attached to a biaxial hydraulic fatigue-testing machine (Instron INC, Norwood, MA) and driven into the bone chips with increasing force (Figure 1). An initial load or set-point of 20–30 N was applied to the proximal impactor in each femur. An initial load of 100 N was then applied at a rate of 5 Hz for 200 impactions on top of this set-point. This load was increased by 100 N every 200 impactions until the femur fractured or until the second mark on the neck of the proximal impactor was reached. If the femur did not fracture, more bone chips were added and the process was repeated. The strain gauges recorded bone surface strain, and Vernier callipers were used to measure cortical thickness along the fracture line and medullary diameter at the origin of the fracture line.

Once the threshold force was determined, a second group of 8 femurs—selected randomly from 8 different cadavers—underwent impaction grafting at a force of 1.6 kN: 4 at an impaction frequency of 1Hz and 4 at an impaction frequency of 10 Hz, and an Exeter no. 1 37.5-mm offset was cemented in each neomedullary canal with a 28-mm or 32-mm head. A force of 1.6 kN was used, as a threshold force of only 0.5 kN was presumed to result in massive implant subsidence on normal weight-bearing forces (440–1,320 N). A force of 1.6 kN was also chosen, as the authors wanted to test the hypothesis that an increased frequency of impaction would lead to less implant subsidence on testing. The bone graft was considered sufficiently compacted when the second mark on the proximal femoral impactor would not subside beyond the femoral neck cut at 1.6 kN of applied force. Thus, the overall number of impactions and impaction duration varied between specimens. The Exeter 37.5-mm offset size-1 stem corresponds to the proximal impactor used.

These constructs were then cemented at their bases on a jig that allowed potting of the constructs at an abduction angle of 7 degrees and a flexion angle of 15 degrees, mimicking the natural alignment of the proximal human femur (Britton and Prendergast 2005). This apparatus did not permit any rotation of the femur.

**Figure 2.**
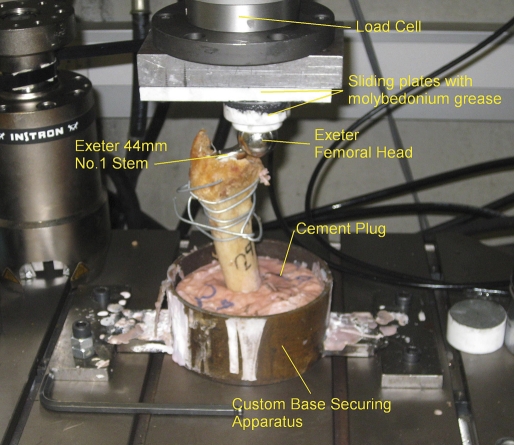
Axial cyclic loading between 440 N (swing phase of gait) and 1,320 N (stance phase of gait) through the 28-mm femoral head, by direct contact with the hydraulic fatigue-testing machine via a PTFE disc that was free to slide along a PTFE plate.

Axial cyclic loading at 440 N (swing phase of gait) to 1,320 N (stance phase of gait) for 150,000 cycles at 3 Hz mimicked the first 2 months of postoperative weight bearing (Figure 2). The position sensor on the Instron machine measured the axial displacement of the femoral head or the subsidence of the construct in the impacted bone graft. Subsidence was defined as the axial displacement between the first and last cycle under 440 N load. During this phase, 2 greased sliding plates allowed the femoral head to move freely at the pressure plate. This technique—to assess stem subsidence following impaction bone grafting—was used by Flannery et al. (2010) in their study on sow femurs.

### Statistics

Regression techniques were used to determine whether a correlation existed between, individually, threshold force and the variables: bone mineral density, cortical thickness, and medullary diameter both on plain radiographs before fracture and direct measurements with Vernier callipers after fracture. Regression techniques were also used to determine whether a correlation existed between impaction frequency and stem subsidence. Correlation coefficients for each suspected association were calculated according to “Statistics at Square One”. (Swinscow 1997). The correlation coefficient can range from values of –1 to +1, with 0 representing no correlation whatsoever between two sets of data. Open Office Suite 3.0 was used to make these calculations.

## Results

**Figure 3.**
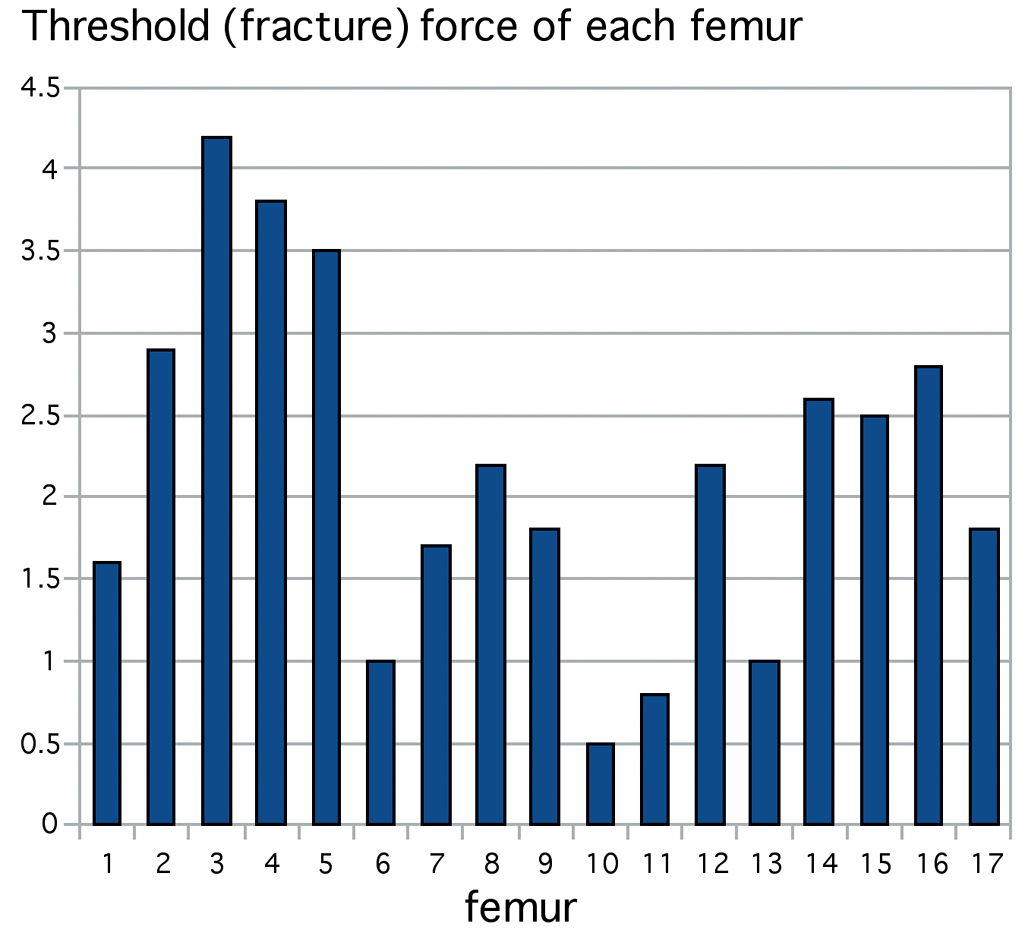
Threshold (fracture) force of each femur in kN.

The threshold force was found to be 0.5 kN (Figure 3). All femurs fractured at a load greater than this. The highest fracture force was 4.2 kN. The average force required to produce fracture was 2.2 (SD 1.1) kN.

The bone surface strain at failure ranged from 880 to 46,000 microstrain, with a limited negative correlation between threshold force and recorded strain that was not statistically significant (correlation coefficient = –0.3). In all cases, the level of the impacted bone was proximal to the lesser trochanter. The average strain recorded at fracture was 8,466 microstrain. There was a correlation between the Gruen zone of maximum recorded strain and the Gruen zone of fracture (correlation coefficient = 0.79), but this did not reach statistical significance (r² = 0.62). In each experiment, the fracture propagated from the proximal femur: 8 from Gruen zone 1 and 9 from Gruen zone 7.

The average, narrowest cortical width at the fracture site was 2.8 (1.4–7.0) mm. The average canal diameter was 29 (22–36) mm and the mean cortex-to-canal ratio was 0.1 (0.04–0.32). There was a positive correlation between threshold force and narrowest cortical diameter at the fracture site (correlation coefficient = 0.55). There was a weak negative correlation between threshold force and medullary diameter at the fracture site (correlation coefficient = –0.37). There was a stronger correlation between threshold force and cortex-to-canal ratio at the fracture site (correlation coefficient = 0.61). None of these correlation coefficients approached statistical significance (r² < 0.7).

There was no correlation between threshold force on the one hand and bi-cortical diameter, medullary cavity diameter, or bi-cortical diameter-to-medullary cavity diameter ratio on the other at the level of the junction between Gruen zones 6 and 7 on plain radiographs before destructive impaction testing. The correlation coefficients were –0.02, –0.09, and 0.02, respectively.

The average BMD was 0.77 (0.53–1.36) g/cm². There was no statistically significant correlation between fracture force and the overall BMD of the femur (correlation coefficient = 0.27, r² = 0.07). There was also no correlation between threshold force and the BMD of the Gruen zone of fracture (correlation coefficient = –0.01). Neither of the above correlation coefficients reached statistical significance (r² > 0.7).

**Figure 4.**
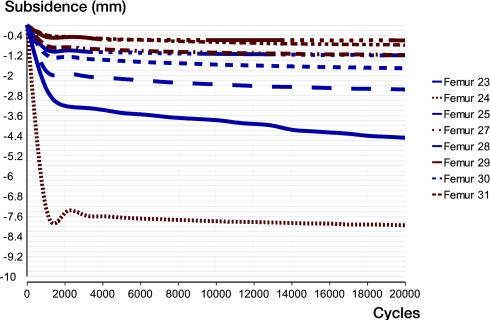
Femoral stem subsidence in the cement mantle (in mm) during the swing phase of gait for each femur over 150,000 cycles, simulating 2 months of postoperative weight bearing. Blue: 10-Hz impaction frequency. Red: 1-Hz impaction frequency. Femur 24, which subsided the most, did not fracture but subsided due to poor cementing technique, resulting in an incomplete cement mantle.

Following impaction with a force of 1.6 kN, the average subsidence for the 8 prostheses after 150,000 cycles was 3.2 (0.95–8.7) mm. There was no correlation between subsidence and the frequency at which the bone graft was impacted (correlation coefficient = 0.04) (Figure 4).

## Discussion

Our experimental goal was to calculate a threshold force for fracture in the embalmed human femur during impaction bone grafting. We used embalmed human femurs due to their easy availability from the anatomy laboratory, with a view to validating the experimental model for further use on fresh human femurs. The formalinized bone probably represented the physical characteristics of the weakened femur in revision hip arthroplasty more accurately. The mechanical properties of embalmed human bone have not been fully elucidated. Two studies on embalmed human cadavers have not reported any gross damage to the macroscopic structure of the cadavers, or gross histological change, which included examination of embalmed cartilage and bone (O' Sullivan and Mitchell 1993, Coleman and Kogan 1998).

Currey et al. (1995) investigated the effect of formaldehyde on the mechanical properties of human bone, reporting quasistatic loading tests that were almost unaffected by fixation but also noting a substantial decrease in impact strength.

Oxland et al. (1995) enzymatically disrupted the collagen crosslinks in rat femora and noted an overall decrease in breaking strength, bending stress, and elastic stiffness of 21%, 26%, and 30%, respectively, compared to rat femurs from the control group. Embalming agents would be expected to have a similar effect on collagen (Asaka and Kikukawa 2005). In an in-vitro study, Oakley and Kuiper (2006) found that washing of the bone graft did not effect the cohesion of the graft particles, indicating that the wet condition in the clinical setting does not influence the properties of the impacted bone graft.

A potential limitation of our study was that there were no cortical defects in the study femurs. Typically, a femur in the clinical setting that is undergoing impaction bone grafting has cortical defects and reduced bone stock. These bony defects would usually be reinforced with cages, meshes, cerclage wiring, and/or strut grafts during the operative procedure.

Further limitations include the removal of the normal soft-tissue envelope during experimentation, which would be expected to provide extra strength to the proximal femur in the clinical setting, suggesting that a threshold force of 0.5 kN may not cause fracture in the clinical setting.

An arbitrary impaction force of 1.6 kN was selected for the second arm of the experiment, as 14 of the initial femurs fractured at or above this level. Impaction of the bone graft at 0.5 kN for the second arm of the study would have resulted in massive implant subsidence, thus not allowing us to demonstrate that if the bone graft is impacted with sufficient force, massive subsidence of the implant will not occur. We suspect that the threshold force for fracture would be higher in fresh, un-embalmed bone—and higher still in the clinical setting with intact soft-tissue envelope and relatively unfixed thigh on the operating table. The maximum force that could be applied through the impaction slap-hammer by an adult male was 3.5 kN. In the clinical setting, the force applied would be much less. This could be measured in the clinical setting by incorporating a load cell into the slap-hammer handle, which would require a redesign of the instrument.

From the previous study on adult sow femurs (Flannery et al. (2010), we did not expect any correlation between bone mineral density, cortical thickness, and canal diameter on the one hand and the threshold force on the other. While correlations between these were found, none reached statistical significance. Surprisingly, no correlation was noted between threshold force, cortical thickness, canal diameter, and cortex-to-canal ratio as measured on standard AP radiographs. Thus, although a non-statistically significant correlation between threshold force, cortex thickness, and canal diameter was demonstrated during destructive testing, we were unable to demonstrate any useful predictive measurements from standard (fully calibrated) pretesting radiographs.

In femoral impaction grafting, the stability of the construct is of great importance in preventing massive early subsidence. Inadequate compaction of the bony chips has been suggested as a cause of massive early subsidence (Eldridge et al. 1997), but overimpaction may increase the femoral fracture rate. The subsidence testing did not show any massive femoral stem subsidence, and we found no correlation between the magnitude of stem subsidence and the frequency of impaction used during impaction bone grafting.

A simple 2-tailed t-test was applied to the results obtained from subsidence testing. A p-value of 0.8 was obtained, strongly suggesting that the subsidence rates at the different frequencies were similar. Due to the small sample size, a type-II error is probable and the authors cannot fully reject the null hypothesis that increased inpaction frequency leads to reduced stem subsidence.

In summary a threshold force of only 0.5 kN was demonstrated in this preliminary study, but with four-fifths of the femurs fracturing above 1.6 kN of applied force. Massive stem subsidence did not occur with an impaction force of 1.6 kN and there was no correlation between magnitude of stem subsidence and the two different impaction frequencies. Unfortunately, no significant correlation between predestructive testing radiographs and DEXA scans and threshold force could be demonstrated. Further testing on fresh human femurs will be necessary to determine a threshold force.
